# Prophylactic Effects of Propranolol versus the Standard Therapy on a New Model of Disuse Osteoporosis in Rats

**DOI:** 10.3797/scipharm.1310-06

**Published:** 2013-12-09

**Authors:** Deepak Kumar Khajuria, Choudhary Disha, Rema Razdan, D. Roy Mahapatra, Ramakrishna Vasireddi

**Affiliations:** 1 Department of Pharmacology, Al-Ameen College of Pharmacy, Bangalore, India.; 2 Department of Aerospace Engineering, Indian Institute of Science, Bangalore, India.

**Keywords:** Propranolol, Immobilization, Rat model, Osteoporosis, Bone strength

## Abstract

Disuse by bed rest, limb immobilization, or space flight causes rapid bone loss by arresting bone formation and accelerating bone resorption. Propranolol (a non-selective β-adrenergic antagonist) has been shown to improve bone properties by increasing bone formation and decreasing bone resorption in an ovariectomy-induced rat model. However, no studies have yet compared the osteoprotective properties of propranolol with well-accepted therapeutic interventions for the treatment and prevention of immobilization/disuse osteoporosis. To clarify this, we investigated the effects of propranolol compared with zoledronic acid and alfacalcidol in a new animal model of immobilization/disuse osteoporosis. Three-month-old male Wistar rats were divided into five groups with six animals in each group: (1) immobilized (IMM) control; (2) normal control; (3) IMM + zoledronic acid (50 μg/kg, intravenous single dose); (4) IMM + alfacalcidol (0.5 μg/kg, per oral daily); (5) IMM + propranolol (0.1 mg/kg, subcutaneously 5 days/week) for 10 weeks. In groups 1 and 3–5, the right hindlimb was immobilized. At the end of treatment, the femurs were removed and tested for bone porosity, bone mechanical properties, and cortical microarchitecture. Treatment with propranolol induced greater reductions in the bone porosity of the right femur and improved the mechanical properties of the femoral mid-shaft femur in comparison to the IMM control. Moreover, treatment with propranolol also improved the microarchitecture of cortical bones when compared with the IMM control, as indicated by scanning electron microscopy. The anti-osteoporotic property of propranolol was comparable with zoledronic acid and alfacalcidol. This study shows that the bone resorption induced by immobilization/disuse in rats can be suppressed by treatment with propranolol.

## Introduction

Osteoporosis is described as a multifactorial disease characterized by low bone mineral mass and deteriorated microarchitecture of the bone tissue leading to enhanced bone fragility and increased risk of debilitating fractures. Immobilization is one of the important causes of osteoporosis. Disuse (unloading) osteoporosis occurs in patients with spinal cord injuries, patients confined to prolonged bed rest, and astronauts exposed to microgravity during space flight. Disuse osteoporosis also occurs in areas of low bone stress around orthopaedic implants and from limb immobilization after surgery [1]. The main clinical manifestation of long-term or short-term immobilization is represented by increased fracture liability [2]. The main feature during immobilization is a dramatic increase in bone resorption and a decrease in bone formation [3]. In spite of continuous and aggressive treatment, immobilization requires a very long time for bone to recover its bone mineral density and mechanical strength [4]. As the goal of today’s medicine shifts more towards disease prevention rather than treatment, prophylactic therapy seems to be the promising choice for disuse osteoporosis.

Zoledronic acid (ZOL), a nitrogen-containing bisphosphonate, is a potent anti-resorptive agent which has been approved and is used for the treatment of various disorders characterized by increased osteoclast-mediated bone resorption due to, for example, estrogen depletion or aging/disuse. ZOL interferes with osteoclastic activity by preventing osteoclast formation and osteoclast-mediated bone resorption activities and by inducing osteoclast apoptotic cell death [5]. Alfacalcidol (ALF) is a prodrug of active vitamin D3, a calcium-regulating hormone which is widely accepted as a baseline treatment for osteoporosis. Supplementation with vitamin D increases muscle strength and thus may reduce the risk of fractures [6]. It has been demonstrated that the administration of vitamin D analogs diminished the effect of immobilization in the development of osteoporosis [7]. Previously, we studied the preventive therapy of administering ALF to rats immediately after an ovariectomy and found that ALF sustained or increased bone mass by suppressing bone resorption while maintaining bone formation [8, 9].

Propranolol (PRO), a non-selective β-adrenergic antagonist, has been shown to improve bone properties in different experimental models of bone disorders [8–13]. Results of some prior epidemiological studies confirm the hypothesis that the use of β-blockers is associated with a decrease in fracture risk [14–16]. According to the available evidence, the β-adrenergic pathway of the sympathetic nervous system is a major transmitter pathway that mediates unloading-induced bone loss through the suppression of bone formation by osteoblasts and the enhancement of resorption by osteoclasts. Studies have demonstrated that PRO could be used to prevent the induced mechanical unloading bone loss [17, 18]. Moreover, studies have demonstrated that low doses of PRO suppress bone resorption by inhibiting the receptor activator of nuclear factor kappa-B ligand (RANKL)-mediated osteoclastogenesis as well as inflammatory markers without affecting haemo-dynamic parameters [13]. This result is supported by a previous finding, which showed that PRO stimulates osteoprotegerin (OPG) on its own in osteoblast cells [17]. PRO has been recommended as one of the first-line treatment drugs for hypertension and has been widely used in cardiovascular disease. Based on the available data, nearly 45% of the aging population suffers from cardiovascular disease and osteoporosis. Therefore, the advantage of a dual-benefit effect with only one treatment for both the heart and skeletal systems is of great interest [9].

With this possibility in mind, the use of PRO was investigated which may lead to a better treatment for the immobilization of osteoporosis when compared to well-accepted therapies such as ZOL and ALF in a rat model of immobilization osteoporosis. A new immobilization model is reported in this study which was very efficient in inducing significant long-term hindlimb disuse.

## Materials and Methods

### Drugs, Chemicals, and Other Materials

ZOL was obtained from Naprod Life Sciences, Maharashtra, India. PRO, ALF, xylene, and ether were obtained from Aurobindo Pharma (Hyderabad, India), Glaxo Smithkline Pharmaceuticals (Mumbai, India), and S.D. Fine Chemicals (Mumbai, India), respectively. Ketamine and xylazine were obtained from Neon Pharma, Mumbai and Indian Immunologicals Ltd., Hyderabad, respectively.

### Pre-Clinical Study Design

Three-month-old male Wistar rats weighing 170–180 g were included in the study. Animals were maintained under controlled temperature at 25 ± 2 °C with a 12 hr light/dark cycle with food and water provided *ad libitum*. The experiments were conducted as per the CPCSEA (Committee for the Purpose of Control and Supervision of Experiments on Animals) guidelines after obtaining ethical clearance from the Institutional Animal Ethical Committee.

### Description of a New Rat Model for Disuse Osteoporosis

**Fig. 1:**
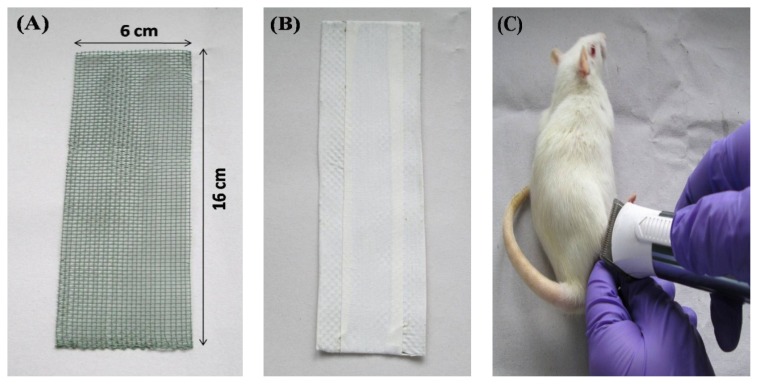

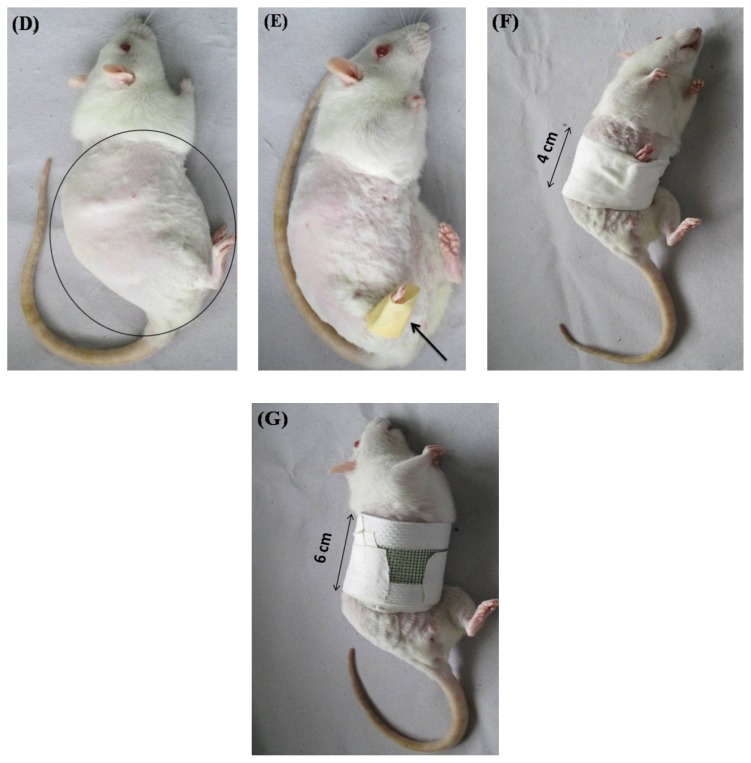
Pictorial representation of the rat hindlimb immobilization method. (A) The frame work is made of PVC-coated iron mesh (measurements in cm), (B) to prevent injury to the animal’s body, PVC-coated iron mesh was covered with microporous adhesive tape (measurements in cm), (C) rat’s lower torso and right hindlimb were trimmed of all hair using an automatic hair trimmer, (D) black circle depicts the trimmed area, (E) protective barrier wipe was applied to the lower right hindlimb (arrow), (F) right hindlimb was immobilized using two layers of microporous adhesive tape, (G) framework encased against the abdomen using microporous adhesive tape (measurements in cm).

The framework for the right hindlimb immobilization of rats was prepared with a durable and high quality PVC (polyvinyl chloride)-coated welded iron mesh. The mesh was 16 cm long and 6 cm wide with a pore size of 1.8 mm, as shown in Fig. 1A. PVC-coated iron mesh was then wrapped with microporous adhesive surgical tape as shown in Fig. 1B, to prevent injury to the animal’s body. The prepared framework can be easily fitted and fixed to a Wistar rat with a body weight of 160–180 grams. Firstly, the reasons for using PVC-coated welded mesh iron mesh were based on being cost-effective, lightweight, soft, suitable for sterilization, and biocompatible. Second and most interestingly, the advantage of this framework was to prevent the animal from destroying the inner microporous adhesive tape, which was wrapped to the animal’s abdomen for immobilizing the hindlimb. To maintain the retention of the framework on the animals, 2–3 layers of microporous adhesive surgical tape (5 cm wide) was placed around the animal’s abdomen. Microporous adhesive tape usually has a hypoallergenic adhesive which is designed to stick firmly to the skin and underlying layers of tape, but removes easily without damaging the skin. Moreover, it allows air to reach the skin. It helps to protect the skin from irritation, abrasion, and infection.

All of the animals were housed singly in cages for 2 weeks for acclimatization to vivarium conditions. Before immobilization of the right hindlimb, the rats were anesthetized with a combination of ketamine (80 mg/kg) and xylazine (10 mg/kg), intraperitoneally. Each animal’s lower torso and right hindlimb were trimmed of all hair using an automatic hair trimmer (Fig. 1C & 1D). A protective barrier wipe was applied to the lower right hindlimb, as shown in Fig. 1E. It provided a barrier film layer on the skin used under the microporous adhesive tape to help protect against irritation, excoriation, and adhesive build-up. The right hindlimb was immobilized against the abdomen with the hip joint in flexion and tibiofemoral joint in extension, using two layers of microporous adhesive surgical tape (4 cm wide, as shown in Fig. 1F). The framework was encased against the abdomen using 6 cm microporous adhesive tape. It was a necessary step to keep the hindlimb in a fixed immobilized position for a longer duration (Fig. 1G). Within 24 h, the rats were able to walk on three legs with no obvious discomfort in locomotion or feeding inside the cage. Throughout the 10-week treatment period the animals were checked daily. Moreover, there was no need to readjust the framework as it was able to immobilize the animal successfully for a longer duration, as compared to the conventional methods, with no indication of skin ulceration, edema, sores, or swelling.

### Experimental Procedure

The rats were divided into five equal groups (n=6). The non-immobilized control group and the immobilized (IMM) control group, served as negative and positive controls, respectively. Normal (non-immobilized) control and IMM groups were subcutaneously administered a vehicle (normal saline, 5 days per week) for 10 weeks. One IMM group was treated with a single intravenous dose of ZOL with 50 μg/kg, administered into the tail vein as a slow intravenous injection over 30 s under light inhalation anaesthesia. One IMM group was orally administered 0.5 μg/kg of ALF daily for 10 weeks. Treatment on the remaining IMM group was initiated with PRO at a dose of 0.1 mg/kg, injected sub-cutaneously 5 days per week, for 10 weeks. The medication dosages used in this experiment were selected from previous studies on a rat osteoporosis model [8, 9]. The dose of ZOL was selected based on the dose-response study carried out in our laboratory.

After 10 weeks of drug exposure or vehicle administration, the animals were humanely sacrificed under anesthesia and the various bone parameters were evaluated.

### Measurement of Femoral Porosity

The femurs of all animals were scanned with the foX-Rayzor, which is a portable X-ray inspection system equipped with “calculate histogram” tool software, according to the method described by Khajuria et al [9]. Briefly, for X-ray analysis, the whole femur was divided into four equal fields, which includes the distal femoral epiphysis (R1), femoral shaft (R2 and R3), and proximal femur (R4).

### Bone Mechanical Tests

Femur strength was assessed by three-point bending as previously described [9]. Briefly, femurs were removed from the −20 °C freezer and rehydrated in a saline solution for 4 h at room temperature. The hydrated weight of the bones was determined using a four decimal place digital scale. The length of the femurs was measured by using a caliper to determine the possible effect on skeleton growth. Specimens were placed on two supports that were separated by a distance of 12 mm and bent until fractured by lowering the crosshead positioned at the mid-shaft at a constant speed of 0.033 mm/s. From the load-displacement curve, the peak load (N), the ultimate stiffness (N/mm), and the toughness (mJ) were obtained. Ultimate stress (strength) and Young’s modulus were derived from the load-deformation curves obtained by using equations described by Khajuria et al [9].

### Cortical Scanning Electron Microscopy

After mechanical strength tests, the fractured surfaces of the right femur were examined by SEM (scanning electron microscopy; Ultra 55, Karl Zeiss Microscopy, Germany) at a magnification of ×600. A proximal-diaphysis section of the right femur of all rats in each group was rendered anorganic by a 5% sodium hypochlorite treatment. The sections were then rinsed in water, dehydrated in acetone, and dried. SEM examinations were carried out for bone pores [19]. Representative SEM photomicrographs were analyzed for the number and size of the pores using image analysis software (Sigma Scan Pro software).

### Statistical Analysis

All data were expressed as the mean ± S.D (standard deviation). For all the data, comparisons between different treatments were analyzed by one-way ANOVA followed by Tukey’s multiple comparison tests. In all cases, a probability error of less than 0.05 was selected as the criterion for statistical significance. Graphs were drawn using Graph Pad Prism (version 5.0 for Windows).

## Results

### Final Body Weight

**Fig. 2:**
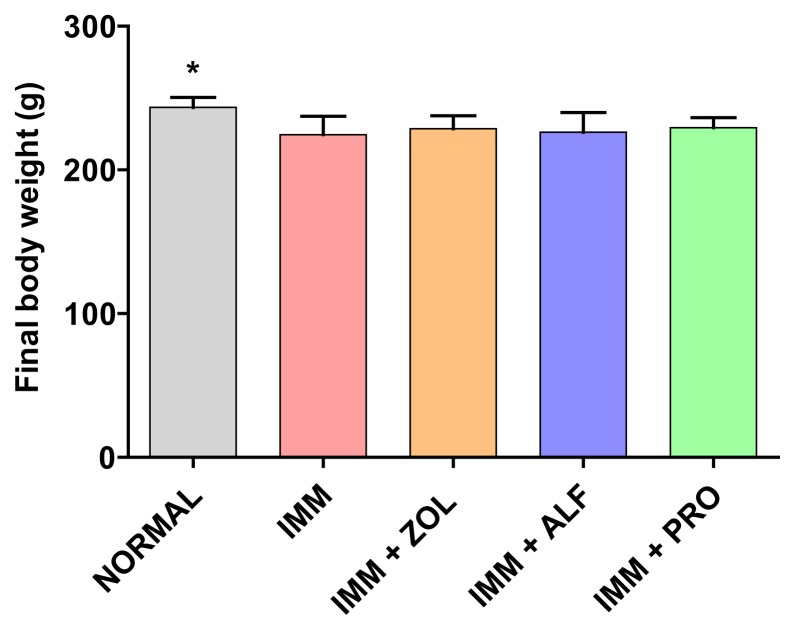
Final body weight of the animals. Data are expressed as the mean ± S.D (n=6), evaluated by one-way ANOVA followed by Tukey’s multiple comparison test. *p < 0.05, compared to IMM control group.

The normal control group had significantly higher body weights than the IMM control group and all treatment groups. There were no statistically significant differences in the weights observed between any of the active treatment groups and that of the IMM control group (Fig. 2).

### Femoral Length

The length of the immobilized femurs was not significantly different from that of the non-immobilized femurs of the same rats in the control group. The administration of all therapeutic interventions did not cause any significant change in the length of the immobilized and non-immobilized femurs (data not shown).

### Effects of Different Treatments on Bone Porosity

#### Immobilized (Right) Leg

**Fig. 3:**
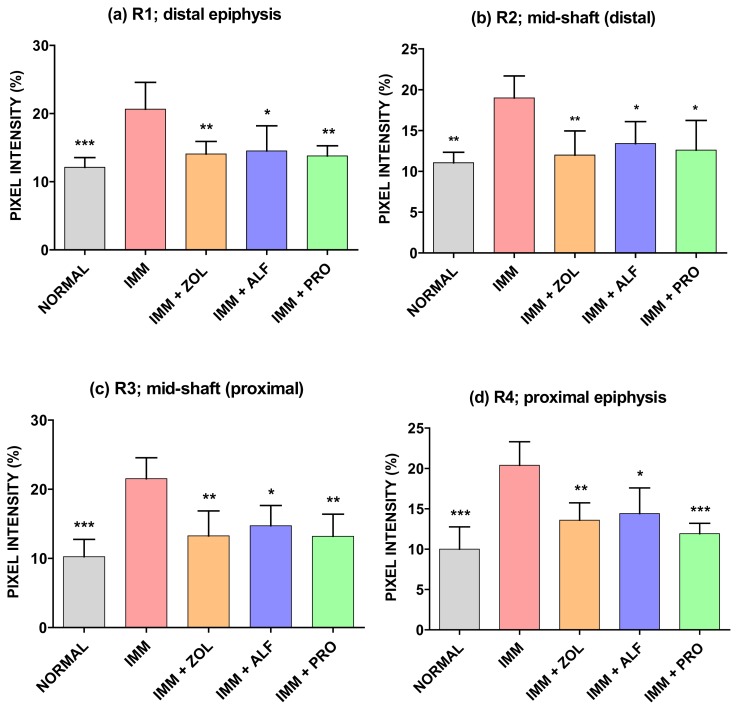
Effects of ZOL, ALF, and PRO on femoral porosity. (a) R1: distal femoral epiphysis, (b) R2: distal femoral shaft, (c) R3: proximal femoral shaft, (d) R4: proximal femoral epiphysis. Data are expressed as the mean ± S.D (n=6), evaluated by one-way ANOVA followed by Tukey’s multiple comparison test. *p < 0.05; **p < 0.01; ***p < 0.001, compared to the IMM control group.

The effects of immobilization and subsequent treatment with ZOL, ALF, and PRO on the porosity of the right femur were measured by X-ray imaging, shown in Fig. 3(A–D). X-ray transmission intensity for the IMM group at R1 (distal epiphysis), R2 (mid-shaft: distal), R3 (mid-shaft: proximal), and R4 (proximal epiphysis) was significantly higher than those for the normal group, which indicated that an immobilization elicited an increase in porosity in these areas. The X-ray transmission intensities of the ZOL, ALF, and PRO groups were significantly lower as compared with the IMM group at R1, R2, R3, and R4. The results of the bone porosity analysis showed no significant difference between all of the pharmacological treatments and the non-immobilized group.

#### Non-Immobilized (Left) Leg

There were no significant differences between groups concerning bone porosity values (data not shown).

### Effect of Different Treatments on Mechanical Properties of the Femoral Mid-Shaft

#### Immobilized (Right) Leg

**Fig. 4:**
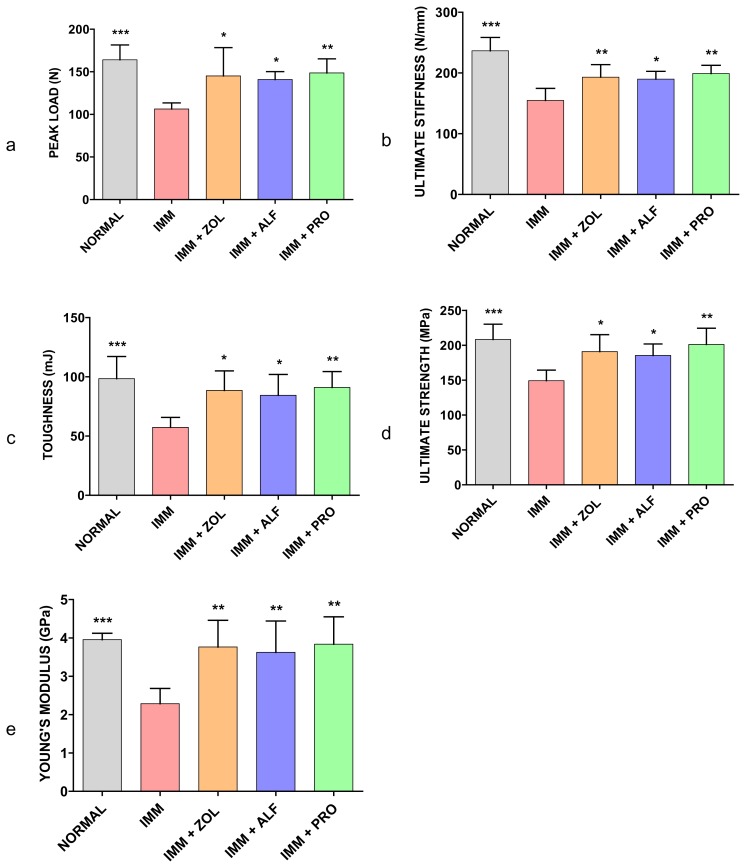
Effects of ZOL, ALF, and PRO on the mechanical strength of the femoral mid-shaft. The femoral mid-shaft was subjected to three-point bending to failure, which provided data on peak load (a), ultimate stiffness (b), toughness (c), ultimate strength (d), and Young’s modulus (e). Data are expressed as the mean ± S.D (n=6), evaluated by one-way ANOVA followed by Tukey’s multiple comparison test.*p < 0.05; **p < 0.01; ***p < 0.001, compared to the IMM group.

Fig. 4(A–E) shows the peak load, ultimate stiffness, fracture toughness, ultimate strength, and Young’s modulus in the femoral mid-shaft, respectively. Three-point bending tests of the right femur indicated that immobilization caused significant reductions in the strength parameters including peak load, ultimate stiffness, fracture toughness, ultimate strength, and Young’s modulus compared with those in the normal or non-immobilized group (*p* < 0.001). The peak load of the femur in the ZOL, ALF, and PRO groups was significantly higher than in the IMM group (*p* < 0.05, *p* < 0.05, and *p* < 0.01, respectively). Moreover, the ultimate stiffness of the femur in the ZOL, ALF, and PRO groups was significantly higher than in the IMM group (*p* < 0.01, *p* < 0.05, and *p* < 0.01, respectively). Also, the fracture toughness of the femur in the ZOL, ALF, and PRO groups was significantly higher than in the IMM group (*p* < 0.05, *p* < 0.05 and *p* < 0.01, respectively). Furthermore, in the ZOL, ALF and PRO groups, the ultimate strength of the femur was significantly higher than in the IMM group (*p* < 0.05, *p* < 0.05, and *p* < 0.01, respectively). The Young’s modulus of the ZOL, ALF, and PRO groups was significantly increased when compared with the IMM group (*p* < 0.01). In contrast, there were no significant differences among each of the treatment groups with respect to peak load, ultimate stiffness, toughness, ultimate strength, and Young’s modulus of the femoral mid-shaft.

#### Non-Immobilized (Left) Leg

There were no significant differences between peak load, ultimate stiffness, fracture toughness, ultimate strength, and Young’s modulus from IMM control animals, normal control animals, or drug-treated animals (data not shown).

### Scanning Electron Microscopy

**Fig. 5:**
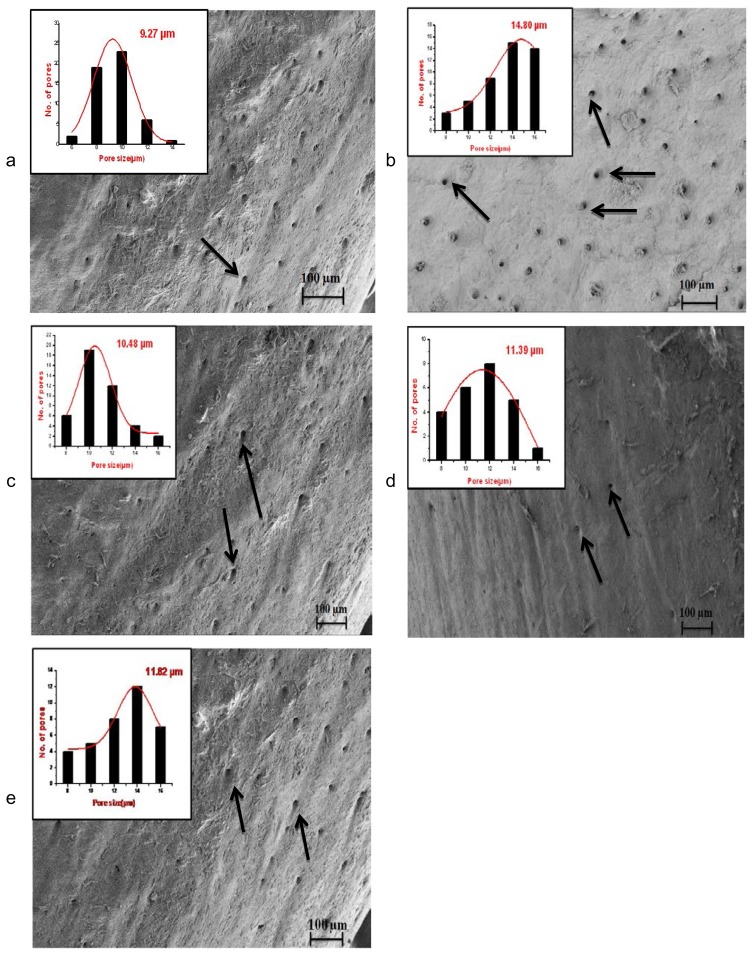
Scanning electron micrographs taken from femoral proximal-diaphysis. Bone pore sites are shown by arrows. The pore geometry image represents the levels of pore diameter.

At the end of the treatment, compared with the normal group, the cortical structure revealed a higher pore diameter (+37.67%, non-significant) and a higher pore number (+41.62%, *p* < 0.01) in the IMM group, which yielded an overall higher cortical porosity +78% (*p* < 0.001). Cortical porosity tended to be lower in ZOL (−70.81%), ALF (−76.95%), and PRO (−79.86%) when compared with the IMM group (*p* < 0.001). Pore number was significantly lower in all treatment groups compared with the IMM group. These results were confirmed by those obtained from the scanning electron micrographs (Fig. 5). Treatment with ZOL, ALF, and PRO improved the microarchitecture of bones.

### Comparison between the Non-Immobilized (Left) Leg and Immobilized (Right) Leg within the Same Group

**Fig. 6:**
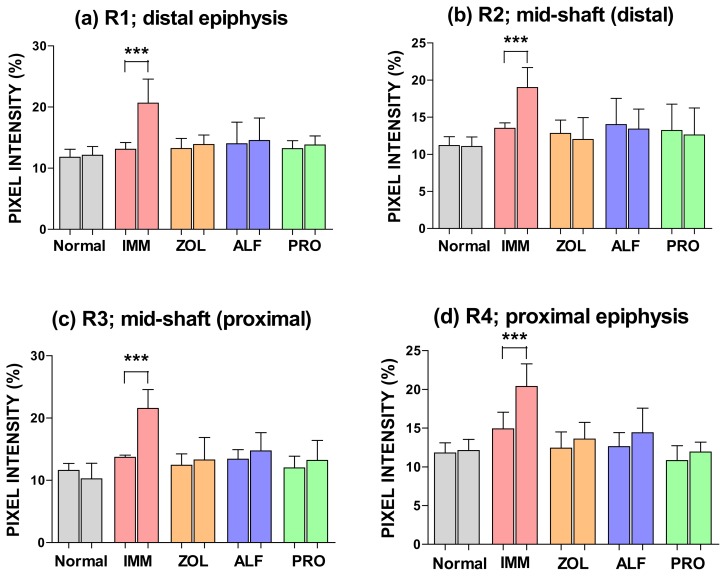
Femoral porosity for the non-immobilized (left bar) and the immobilized (right bar) side within the same group. Asterisk denotes significant difference between the non-immobilized side and the immobilized side (mean ± SD).

**Fig. 7:**
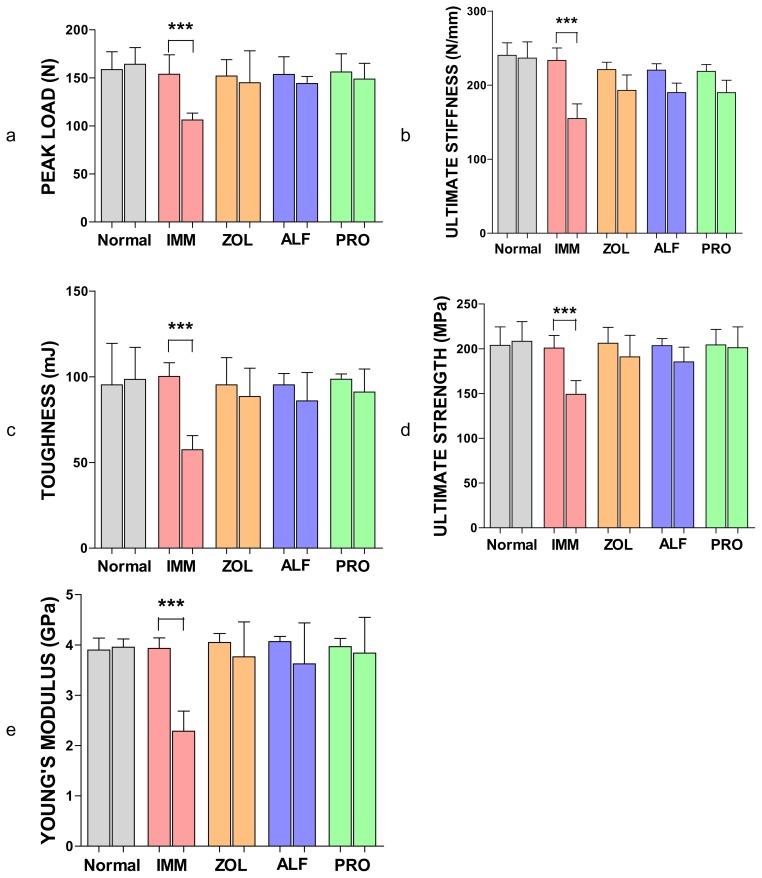
Mechanical properties for the non-immobilized (left bar) and the immobilized (right bar) side within the same group. The femoral mid-shaft was subjected to three-point bending to failure, which provided data on peak load (a), ultimate stiffness (b), toughness (c), ultimate strength (d), and Young’s modulus (e). The asterisk denotes significant differences between the non-immobilized side and the immobilized side (mean ± SD).

The bone porosity and mechanical properties of the left and right legs are plotted as “split-bar” diagrams in Fig. 6 & 7, respectively. An asterisk indicates that there was a significant difference between the left and right leg within the same group (Wilcoxon’s signed-rank test). At R1, R2, R3, and R4 regions, the X-ray transmission intensity for the immobilized side (right) seemed significantly higher than those from the non-immobilized side (left) in the IMM group (*p* < 0.01). In contrast, treatment with the standard therapy or PRO showed full protection against disuse osteoporosis at R1, R2, R3, and R4 regions, as indicated by X-ray transmission intensity values (Fig. 6).

At the femoral mid-diaphysis (three-point bending test), the effect of immobilization was very pronounced in the IMM group; that is, the immobilized side (right) had significantly lower values of strength parameters including peak load, ultimate stiffness, fracture toughness, ultimate strength, and Young’s modulus than the non-immobilized side (left) (*p* < 0.01). Treatment with all therapeutic interventions showed full protection against immobilization (Fig. 7).

## Discussion

A number of animal models for studying the influence of disuse on bone have been proposed [20]. Immobilization of the hindlimb of rats has been repeatedly used as an animal model to investigate disuse osteoporosis. In laboratory animals, localized disuse of a single extremity can be obtained by different ways: denervation, bone resection, tenotomy, devascularization, or arthrodesis. However, it has been shown that bone loss results from the cumulative effects of the disuse and the regional acceleratory phenomenon caused by the surgical trauma. A non-surgical method like transient muscle paralysis is caused by the botulinum toxin, which has been found to provoke rapid bone loss with the cessation of active muscle contraction [21]. A minute quantity of the botulinum toxin may spread to adjacent tissues or enter the circulatory system. Due to this diffusion, it can produce regional or systemic side effects [22]. Other non-surgical methods such as immobilization by casting, bandaging, or tail suspension are preferred because they do not expose the subject to surgical trauma. The need for special cages, difficulties in obtaining correct and reliable bandaging, the necessity to regularly reapply the tape bandaging (one or two times per week to maintain immobilization), the necessary redistribution of bone strains due to non-physiological positions, and the muscle compression created in these models limit their interest [20, 23, 24]. Studies conducted in our laboratory showed that immobilization made of casts were too heavy (50–60% of the rat weight), so there was difficulty in the handling and restraint of the rat during the period of immobilization. Despite the recognized effectiveness of casts in inducing hindlimb immobilization, they must be adjusted to each animal for optimal results, which is very time-consuming. Moreover, a thin layer of padding and a layer of fiberglass must be placed beneath the cast for preventing dermatitis and preventing the animal from chewing through the cast, respectively. Furthermore, the rat must be monitored on a daily basis for fecal clearance, chewed plaster, abrasions, venous occlusion, and problems with ambulation, which may require the readjustment and/or reinforcement of the cast. Other difficulties with this technique of immobilization involve a series of complicating factors, i.e difficulty in observing the rat skin and in some cases, the rat skin showed skin ulcerations or swelling probably due to the retention of urine by the cast. In some cases, rats showed marked weight loss due to fixed immobilization and development of edema in the distal extremity of the immobilized limb. Moreover, some rats were able to slip out of the immobilization. Since cast immobilization is a major experimental challenge, the development of a convenient, easy-to-perform immobilization procedure will significantly facilitate this important area of research.

After several tests trying to find an alternative method for immobilization to avoid the problems caused by a cast, we developed a new model which is described in this research paper. The new immobilization model adopted in the present study has shown that in rats, 10 weeks of right hindlimb immobilization caused a very pronounced loss of bone. In the data obtained in preliminary studies carried out in our laboratory, the smaller weight of the framework compared with a plaster cast kept the difficulty in movement and locomotion to a minimum, with a consequent minimal body weight loss throughout the period of immobilization. Moreover, no skin ulceration or foot swelling was found in the animals when the immobilization was removed.

Body weight in the normal group was greater than in the IMM group. This indicates that the decrease in body weight of the IMM group was caused by right hindlimb immobilization. Earlier studies by others have shown a similar decrease in body weight after immobilization [2, 25].

Kondo et al indicated that sympathetic tones regulate the immobilization-induced enhancement in bone resorption. This was evidenced by the observations that the inhibition of sympathetic tones by propranolol suppressed immobilization-induced bone resorption, and this led to suppression in bone loss [17]. As expected, PRO administered for 10 weeks reduced the osteopenia induced by immobilization, as demonstrated by the improvements in bone properties in immobilized long bones. Interestingly, immobilization of the right leg also induced a small decrease in bone properties of the non-immobilized contralateral leg, most likely because the immobilized animals did not use that leg as much as the control animals. Moreover, in the present study, osteoporosis induced by immobilization did not result from alterations in skeletal growth because body growth and longitudinal femur length in the immobilized limb were not altered.

A microarchitectural observation of the fractured surfaces of the femur (proximal-diaphysis) was performed with SEM. Results of the SEM analysis showed poor a microarchitecture of the bone (femur) with an increase in cortical porosity in the IMM control animals as against the normal control. Rats treated with PRO had lower cortical porosity, pore number, and higher space between pores compared with the IMM group. The current data correlate with findings from a previous study conducted by Bonnet et al [19], demonstrating the effects of PRO on cortical bone properties.

In this study, a prominent increase in porosity was observed at R1 (femoral distal epiphysis), R2 (mid-shaft: distal), R3 (femoral mid-shaft: proximal), and R4 (femoral proximal epiphysis), after immobilization. The increase in the bone porosity at the R1–R4 regions of rat femoral bone due to unloading was suppressed by the treatment with PRO. Similarly, in the ZOL and ALF groups, the protective effect on femoral porosity was observed at the R1, R2, R3, and R4 regions. The results of bone porosity found by X-ray imaging were consistent with those of microarchitectural observation of the fractured surfaces of the femur (proximal-diaphysis) by SEM.

Unloading induces rapid bone loss and increases fracture risk significantly, especially in elderly bedridden patients. In this experiment, the mechanical properties of the rat femoral bone decreased in the IMM group when compared to the normal group, suggesting an increase in the fragility of the cortical bone of IMM rats. PRO was capable of maintaining bone strength at a normal level (not significantly different from the non-immobilized leg). Similarly, ZOL and PRO maintained bone strength at the distal femoral metaphysis. It should be noted, though, that there were no significant differences in various bone parameter values in a direct comparison between PRO, ZOL, and ALF. The current data correlate with findings from our previous study on PRO, demonstrating the effects of PRO on the mechanical properties of ovariectomized rat bone [9].

The present study has also clearly demonstrated that the two standard drugs, ZOL and ALF, each effectively protected against immobilization-induced bone loss. ZOL and ALF were capable of maintaining bone porosity, cortical microarchitecture, and bone strength at a normal level (not significantly different from the non-immobilized leg). ZOL therapy, which is the popular regime for osteoporosis, is associated with an increased risk of osteonecrosis of jaw, atrial fibrillation, atypical fracture, and renal toxicity. Similarly, treatment with ALF is associated with a higher risk of hypercalcemia and hypercalciuria [6]. Studies have demonstrated that heart hemodynamic functions were preserved under 0.1 mg/kg/day dose of PRO treatment. Cardiac output and other echocardiographic assessments were unaffected by low doses of PRO, the same dose that prevented bone loss [10, 13]. If this is the case, PRO, with little known toxicity, would make an excellent candidate for the therapeutic prevention and treatment of disuse osteoporosis.

## Conclusion

In summary, the immobilization framework proposed in this study was effective in producing long-term disuse in the hindlimbs of rats and is a good alternative to the traditional methods of immobilization. The present study tentatively suggests that PRO is an effective treatment for immobilization osteoporosis. The findings are consistent with the effects of PRO on estrogen deficiency bone loss and extend our knowledge regarding the effects of this therapy in immobilization-induced bone loss. The present study indicates that PRO may play an important role in the clinical management of osteoporosis and similar disorders.
